# Artificial intelligence accelerates efficient mining of functional peptides

**DOI:** 10.1093/lifemedi/lnad005

**Published:** 2023-02-23

**Authors:** Xudong Zhang, Cesar de la Fuente-Nunez, Jun Wang

**Affiliations:** CAS Key Laboratory of Pathogenic Microbiology and Immunology, Institute of Microbiology, Chinese Academy of Sciences, Beijing 100101, China; University of Chinese Academy of Sciences, Beijing 100049, China; Machine Biology Group, Departments of Psychiatry and Microbiology, Institute for Biomedical Informatics, Institute for Translational Medicine and Therapeutics, Perelman School of Medicine, University of Pennsylvania, Philadelphia, PA 19104, USA; Departments of Bioengineering and Chemical and Biomolecular Engineering, School of Engineering and Applied Science, University of Pennsylvania, Philadelphia, PA 19104, USA; Penn Institute for Computational Science, University of Pennsylvania, Philadelphia, PA 19104, USA; CAS Key Laboratory of Pathogenic Microbiology and Immunology, Institute of Microbiology, Chinese Academy of Sciences, Beijing 100101, China; University of Chinese Academy of Sciences, Beijing 100049, China

**Figure 1.**
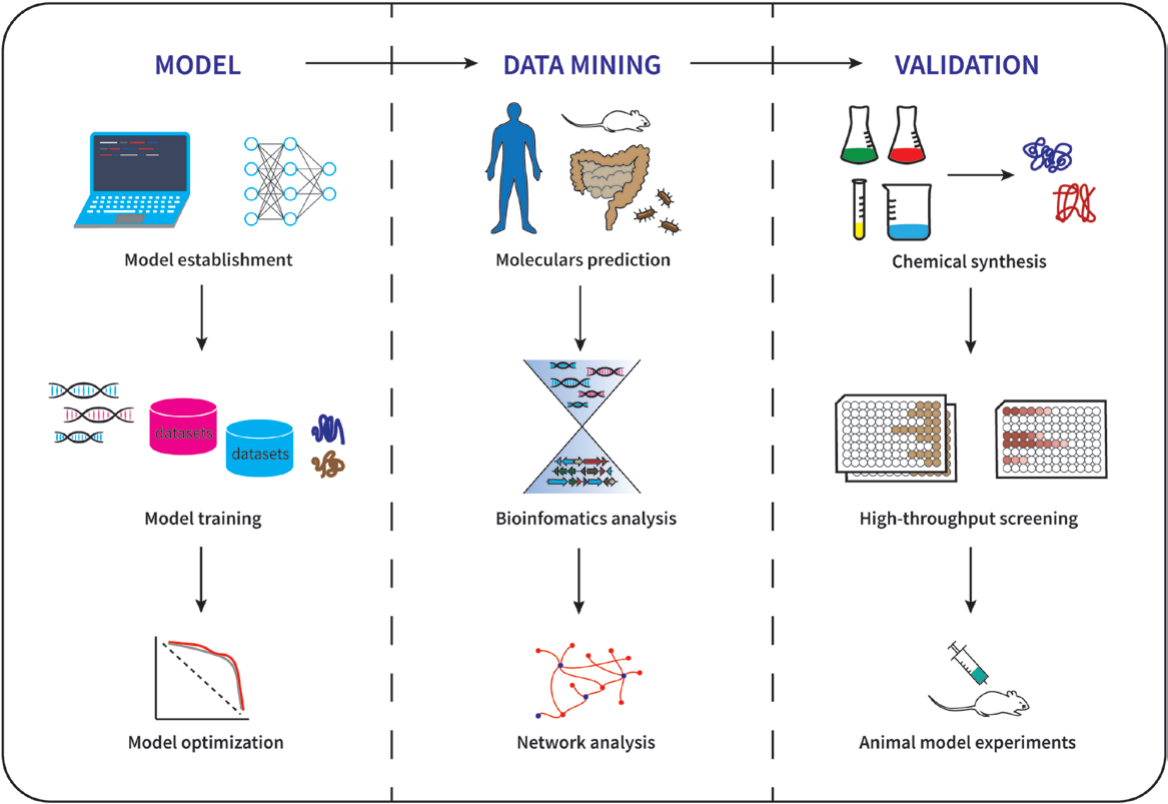
Artificial intelligence guided discovery strategy of functional molecules. Models for molecular prediction are first constructed, and existing datasets are used for training and optimization. With the assistance of bioinformatics analytics, target biomolecules are mined in proteomes, genomes, and metagenomes. Finally, predicted molecules are chemically synthesized and validated, with their mechanism in animal models investigated to verify the potential usage of the molecules.

Deep learning has made remarkable progress in recent years, attracting substantial attention in both biology and medicine. The capabilities of deep learning greatly surpass traditional approaches, and it is beginning to demonstrate success and open new perspectives for drug discovery (Fig. 1). A number of research groups have carried out discovery projects on small functional molecules through deep learning, sometimes in combination with other computational methods. For example, a deep neural network capable of predicting molecules with antimicrobial activity has been applied to discover halicin, which displays bactericidal activity against a wide range of bacterial pathogens [1]. Another deep generative model, termed generative tensorial reinforcement learning, has been used for *de novo* small-molecule design and to discover potent inhibitors of the discoidin domain receptor 1 [2]. Previously, a study described peptide antibiotics designed using computers through evolutionary programming, which displayed potent antimicrobial activity *in vitro* and anti-infective efficacy in a mouse model. Deep learning and computational tools are thus accelerating the design and discovery of new compounds while facilitating the mining of potential target small molecules from datasets.

Antibiotics and their discovery are life-saving medicines that represent one of the greatest innovations of human medicine. However, the overuse and misuse of antibiotics has led to the development and spread of resistance in pathogenic bacteria, leading to so-called superbugs which constitute a major threat to global health. The emergence of antibiotic resistance out-paces the development of novel and effective antibiotics, resulting in an even higher demand for discovery of alternative antibacterial drugs. In this respect, antimicrobial peptides (AMPs) are considered as potential complementary or substitute therapies for alleviating antibiotic resistance [3]. AMPs are capable of microbial cell lysis or growth arrest and are encoded throughout the tree of life including in archaea, bacteria, fungi, plants, and animals. Moreover, they possess desired therapeutic properties including decreased likelihood of resistance-development and transfer. While the discovery of novel AMPs is highly desirable, it is difficult as it relies heavily on time consuming and costly experimental approaches, requiring the development of new methods such as deep learning and the utilization of large-scale omics datasets.

Two recent studies have highlighted the power of artificial intelligence (AI) to discover functional macromolecules, in this case AMPs from different protein collections. By treating protein sequences as functions embedded in sequences, true AMPs and false (nonfunctional) peptides can be distinguished from features deeply hidden within their amino acid composition and relative compositions, instead of one-dimensional sequence similarities. First, after traditional discovery of AMPs and pilot work in combining them with computational methods, Torres et al. [4] reported the first proteome-wide exploration of the human body, which led to the identification of numerous peptides encoded within human proteins. The method mined the human proteome and deliberately avoided using previously described conserved AMP amino acid sequences. Instead, the algorithm searched for AMPs based on their sequence length, net charge, and average hydrophobicity. The peptides identified are encoded within larger proteins that do not appear to be linked to the immune system. Among the hundreds of millions of possible peptides within protein sequences, the study identified 2,603 potential AMPs. In order to validate their prediction, Torres et al. synthesized 56 representative peptides and characterized them in detail. The amino acid patterns, as well as the physicochemical properties of these potential AMPs were significantly different from those in existing peptide databases. These novel putative AMPs may represent a new class of natural peptide antibiotics that play a role in host immunity. Further, by synthesizing a library composed of encrypted peptides and evaluating their antibacterial ability against eight clinically relevant pathogens, a majority of the tested encrypted peptides showed antibacterial activity against relevant bacterial pathogens, demonstrating the reliability of the employed algorithm.

To assess the development of resistance, the above study then performed resistance induction assays with peptides derived from CUB domains 1 and 3 against *Acinetobacter baumannii*, using polymyxin B as an antibiotic control. The results demonstrated that the encrypted peptides have different mechanisms of action from polymyxin B and did not induce resistance during the experiments. Importantly, additional mechanistic assays indicated that the mode of action of the encrypted peptides involved targeting the outer membrane. Animal infection models were used to determine the anti-infective efficacy of the peptides, demonstrating their promising properties as candidates for further antimicrobial agent development. To summarize, using the human proteome as a novel source for data mining, AI approaches may be used to identify novel encrypted peptides with potent antimicrobial properties. As these peptides are derived from human protein, they are likely to be safe for use in humans.

Following this study, the discovery of AMPs in human microbiome data was also reported [5]. Ma et al. focused on the gut microbial metagenome, in which many potential proteins, including AMPs, are encoded. This AMP prediction model was constructed through combination of natural language processing algorithms of three major categories of neural networks. By optimizing the performance of the model, a unified pipeline for AMP recognition was formed. In the process of optimization, it was discovered that the prediction biases of several models were independent of each other, and combining different models further improved the accuracy to 91.31%, with a recall of 83.32%. Further mining of large-scale human microbiome data (4,409 representative genomes and more than 10,000 metagenomic samples) identified 2,349 candidate AMPs with a length distribution of 6–50 amino acids. A total of 241 AMPs were filtered based on association network analysis in bacterial species, 216 were chemically synthesized and 181 showed antibacterial activity, with a positive rate of 83%. The homology of the newly discovered AMPs and the AMPs present in the training set was <40%, and structural similarities were also low, revealing discovery of novel peptides through this approach.

The above study further selected the most potent AMPs based on their activity spectrum and minimal inhibitory concentrations. Of the 10 most potent AMPs, a majority of them were effective even against multi-drug resistant strains included in the ESKAPE list of antibiotic-resistant bacteria of concern. Based on cellular assays, three peptides were found to be nontoxic against human cell lines and did not lead to red blood cell lysis. In a mouse model of bacterial pulmonary infection, the three selected AMPs reduced bacterial load by more than 10-fold, accelerating the recovery of mice after infection. The study also investigated the mechanism of action of the peptides and found mostly canonical sites of action such as cell membrane and cell walls, though a few might have new mechanisms that the assays were unable to uncover. In short, the study mined AMPs from a large reservoir of functional proteins starting with metagenomic sequences, and found a higher number of candidates to filter in each step, eventually identifying AMPs that are functional *in vivo* for the bacterial infection treatment.

The application of deep learning and AI methods in the field of molecular discovery has already demonstrated unprecedented success in terms of throughput and novelty of molecules. Moving forward, the increased prevalence of proteomics, genomics, and transcriptomics datasets are likely to lead to higher rates of discovery. On the other hand, successful deep learning approaches require sufficient training data to build and optimize models. From a methodological perspective, identifying single molecules with a particular function is already feasible, but protein complexes or pathways that involve multiple members remain difficult to identify. Finally, the collection of natural proteins represents only a small portion of the entire sequence space of all possible protein sequences, leaving a wide array of sequences to be computationally discovered. Artificial peptides and proteins may be identified in the future to help address present-day problems. We envision that other biological molecule types, such as DNA, RNA, and oligosaccharides may be mined as functional molecules using deep learning and AI approaches. We anticipate that this emerging field of AI for drug discovery will lead to exciting new approaches to eventually develop much-needed medicines.
